# A New Antiseptic

**Published:** 1898-09

**Authors:** 


A New Antiseptic. Sulpho-carbolate of silver occurs in small prismatic crystals, colorless and odorless, and contains a trifle more than 28 per cent. of silver. It is an efficient antiseptic, and in ophthalmic practice is to be preferred to the nitrate on account of its greater solubility and non-caustic properties.

